# Within-session propulsion asymmetry changes have a limited effect on gait asymmetry post-stroke

**DOI:** 10.21203/rs.3.rs-5053605/v1

**Published:** 2024-12-23

**Authors:** Sarah A. Kettlety, James M Finley, Kristan A. Leech

**Affiliations:** University of Southern California; University of Southern California; University of Southern California

**Keywords:** Stroke, propulsion, asymmetry, walking, combined gait asymmetry metric

## Abstract

Biomechanical gait impairments, such as reduced paretic propulsion, are common post-stroke. Studies have used biofeedback to increase paretic propulsion and reduce propulsion asymmetry, but it is unclear if these changes impact overall gait asymmetry. There is an implicit assumption that reducing propulsion asymmetry will improve overall gait symmetry, as paretic propulsion has been related to numerous biomechanical impairments. However, no work has investigated the impact of reducing propulsion asymmetry on overall gait asymmetry. We aimed to understand how within-session changes in propulsion asymmetry affect overall gait asymmetry, operationalized as the combined gait asymmetry metric (CGAM). We hypothesized that decreasing propulsion asymmetry would reduce CGAM. *Methods.* Participants completed twenty minutes of biofeedback training designed to increase paretic propulsion. We calculated the change in propulsion asymmetry magnitude (Δ|PA|) and the change in CGAM (ΔCGAM) during biofeedback relative to baseline. Then, we fit a robust linear mixed-effects model with ΔCGAM as the outcome and a fixed effect for Δ|PA|. *Results.* We found a positive association between Δ|PA| and ΔCGAM (β = 2.6, p = 0.002). The average Δ|PA| was − 0.09, suggesting that, on average, we would expect a CGAM change of 0.2, which is 0.5% of the average baseline CGAM value. *Conclusions.* Reducing propulsive asymmetry using biofeedback is unlikely to produce substantial reductions in overall gait asymmetry, suggesting that biofeedback-based approaches to reduce propulsion asymmetry may need to be combined with other interventions to improve overall gait asymmetry. *Clinical Trial Registration*. NCT04411303.

## BACKGROUND

Individuals post-stroke commonly present with biomechanical gait impairments such as increased spatiotemporal asymmetries, reduced paretic propulsion, and reduced swing knee flexion.^1,2^ Paretic propulsion is a popular target for clinical interventions and research studies because it is associated with walking speed in individuals post-stroke^3–6^ and can be increased using a variety of interventions (gait biofeedback,^7^ functional electrical stimulation,^8,9^ robotics,^10^ etc.). Additionally, repeated training targeting paretic propulsion can improve functional balance, walking speed,^8^ and cost of transport.^11^ Research studies investigating these interventions mainly focus on quantifying the changes in paretic propulsion or propulsion asymmetry and do not consider whether changes in paretic propulsion impact symmetry in other gait impairments. The assessment of stroke survivor stakeholder values indicates that overall gait asymmetry (i.e., gait appearance) is a priority to address during rehabilitation,^12^ making it important to understand how an intervention impacts overall gait asymmetry.

There is an implicit assumption that reducing propulsion asymmetry will result in improved symmetry in the entire gait pattern, as paretic propulsion has been related to numerous biomechanical impairments such as paretic knee flexion,^13^ paretic and non-paretic trailing limb angle,^14^ and paretic step length.^3^ However, no work has directly investigated the impact of reducing propulsion asymmetry on overall gait asymmetry. With numerous degrees of freedom in the lower limb, it is possible that reducing propulsion asymmetry may not reduce other biomechanical impairments and, therefore, may not improve overall gait asymmetry.

The primary aim of this study was to understand how within-session changes in propulsion asymmetry affect overall gait asymmetry in individuals with chronic stroke, operationalized by the combined gait asymmetry metric (CGAM).^15,16^ The CGAM provides a single comprehensive measure of overall kinematic and spatiotemporal gait asymmetry (bounded between 0–200), allowing for the inclusion of any biomechanical impairment.^15,16^ With CGAM, we can assess the impact of changing propulsion asymmetry on overall gait asymmetry, not just on a single biomechanical impairment. To manipulate propulsion asymmetry, we used visual biofeedback to increase paretic propulsion. Because of paretic propulsion’s relationship with numerous biomechanical impairments,^3,13,14^ we hypothesized that a decrease in propulsion asymmetry would reduce CGAM.

## METHODS

### Participants

Twenty-nine participants at least six months post-stroke completed a single session of paretic propulsion biofeedback training. Participants were recruited from the community and the Registry for Aging and Rehabilitation Evaluation database at the University of Southern California. Participants were included if they could walk independently for five minutes on a treadmill, had paresis confined to one side, were aged 18–80, and had no exercise contraindications. Participants were excluded if they had damage to the pons, basal ganglia, or cerebellum on an MRI, signs of cerebellar involvement or extrapyramidal symptoms, uncontrolled hypertension, orthopedic or pain conditions, or Montreal Cognitive Assessment five-minute protocol score less than nineteen. If a participant regularly wore an ankle-foot orthosis for community ambulation, they wore it during the study.

### Clinical assessments

We assessed motor impairment using the lower-extremity Fugl-Meyer scale^17^ and balance using the Berg Balance Test.^18^ We measured cardiovascular endurance using the six-minute walk test^19^ and determined overground self-selected gait speed using the ten-meter walk test.^20^

### Experimental protocol

This protocol is described in detail in the text of Supplemental Methods. Briefly, we collected kinematic and kinetic data while participants walked on an instrumented treadmill at their self-selected speed. First, participants walked for two minutes without biofeedback. Then, they completed four five-minute biofeedback trials (see Supplemental Fig. 1A, for experimental paradigm). During the biofeedback trials, participants walked with paretic propulsion biofeedback, which provided the paretic limb’s real-time anterior ground reaction force during stance. After each stride, participants were also given feedback of their peak paretic propulsion. The biofeedback goal was the midpoint between peak paretic and non-paretic propulsion at baseline,^7^ with a ± 5N goal zone. Please see Supplemental Fig. 1C for a schematic of the biofeedback paradigm. Some participants used the handrails (light touch) during the experiment for balance aid (n = 11).

Kinematic data were acquired using a ten-camera motion capture system (Qualisys AB, Goteborg, Sweden; 100 Hz), and kinetic data using a dual-belt instrumented treadmill (Bertec Corporation, Columbus, OH, USA; 1000 Hz). Participants wore a harness for safety; however, the harness did not provide body weight support. Markers were placed bilaterally on the iliac crest, greater trochanter, lateral femoral epicondyle, lateral malleolus, and fifth metatarsal head.

### Data processing

All data were processed and analyzed in MATLAB R2020a (MathWorks, Natick, MA). Kinematic data were low-pass filtered with a 6 Hz cutoff, and kinetic data were low-pass filtered with a 20 Hz cutoff.^21^ Foot-strike and toe-off were estimated as the most anterior and posterior positions of the lateral malleoli markers, respectively.

We first calculated peak propulsion (P) as the most positive value of the anterior/posterior ground reaction force during the stance phase for both limbs. Positive values corresponded to anteriorly directed ground reaction forces. Then, we calculated propulsion asymmetry magnitude (|PA|; Eq. 1).^22^

1 $$|\text{PA}|=\frac{abs\left(P_{r}-P_{l}\right)}{P_{r}+P_{l}}$$

We then calculated the change in propulsion asymmetry magnitude (Δ|PA|) from baseline for each stride taken during the biofeedback trials. To do this, we averaged propulsion asymmetry magnitude across the final thirty strides of the baseline trial. Then, we subtracted this value from propulsion asymmetry magnitude for each stride taken during the biofeedback trials, reflecting how much participants changed propulsion asymmetry compared to baseline.

### Combined Gait Asymmetry Metric

The CGAM is an overall gait asymmetry measure that allows for the inclusion of any lateralized biomechanical measures of interest and provides a single measure of overall gait asymmetry between 0 (no asymmetry) and 200 (completely asymmetric).^15^ First, a symmetry index is calculated for each biomechanical impairment of interest using Eq. 2. Then, the symmetry indices $\left(s_{i}\right)$ are combined into a single symmetry matrix for each participant (S), with m columns (number of metrics) and n rows (number of strides). Finally, the symmetry matrix and the covariance of the symmetry matrix $\left(K_{S}\right)$ are used to calculate CGAM for each stride (Eq. 3).

2 $$s_{i}=100*\frac{abs\left(M_{right}-M_{left}\right)}{0.5\;\left(M_{right}+M_{left}\right)}$$

3 $$CGAM=\sqrt{\frac{S*inv(K_{s})*S\prime }{\sum inv\;(K_{s})}}$$

We had eight candidate variables to include in the CGAM calculation: single-limb support time, double-limb support time, stance time, step length, peak swing knee flexion, peak hip flexion, trailing limb angle, and circumduction. Before calculating CGAM using these variables, we checked for collinearity using a variance inflation factor (VIF) cutoff of ten^23^ to ensure we were not including variables that were closely related. We found that stance time and single-limb support time had VIF values greater than ten. After removing stance time, the remaining variables had a VIF of less than ten. Therefore, we included the following seven variables in the CGAM calculation: double-limb support time, single-limb support time, step length, peak swing knee flexion, peak hip flexion, trailing limb angle, and circumduction. Definitions for the variables included in the CGAM calculation are described in the following section. We chose not to include propulsion in the CGAM calculation to understand the effect of manipulating propulsion asymmetry on overall gait asymmetry outside of what the biofeedback was designed to change.

Finally, we calculated the change in CGAM (Δ CGAM) from baseline for each stride. To do this, we averaged the CGAM values from the final thirty strides of the baseline trial and subtracted this value from the CGAM of each stride taken during the biofeedback trials.

### Definitions of variables included in CGAM calculation

Step length was defined as the anterior-posterior distance between the lateral malleoli markers at heel strike. Knee flexion was defined as the angle between the thigh segment (between lateral tibial epicondyle to greater trochanter markers) and shank segment (between lateral malleolus and lateral tibial epicondyle) in the sagittal plane. Hip flexion was defined as the sagittal plane angle between the thigh and pelvis segments (between greater trochanter and iliac crest markers). Circumduction was the maximal lateral difference between the ankle marker during swing and the same ankle marker during stance. Trailing limb angle was defined as the angle between the vertical lab axis and the vector created by the greater trochanter and lateral malleoli markers in the sagittal plane. Double-limb support time was the time from contralateral heel strike to ipsilateral toe off. Single-limb support time was the time from the contralateral toe-off to the contralateral heel strike.

### Statistical analyses

All statistical analyses were performed in R (4.2.2).^24^ First, we confirmed that all participants had propulsion asymmetry at baseline. Participants with a baseline propulsion asymmetry < 0.11 (minimal detectable change for propulsion asymmetry in our data), were removed from all analyses. Then, we wanted to establish that participants used biofeedback to increase paretic propulsion as intended. To do this, we first normalized paretic propulsion to body weight and then averaged it over the final thirty strides of each trial. Then, we fit a linear mixed effects model with average paretic propulsion as the outcome, a fixed effect for trial, and a random intercept using the lme4 package.^25^

Next, we determined how increasing paretic propulsion influenced propulsion asymmetry by fitting a linear mixed-effects model with Δ|PA| as the outcome, a fixed effect for change in paretic propulsion, and a random intercept for each participant. We also included a random slope because it improved the model fit (determined using the Bayesian information criterion score). We fit the model to the final 113 strides of each biofeedback trial for each participant, which matched the minimum number of strides per biofeedback trial taken in the cohort, ensuring that each participant had data from the same number of strides included in the analyses. After fitting the model, the residuals did not meet regression assumptions (checked using the performance package^26^); therefore, we ran robust mixed-effects models to account for those violations^27^ and report those results.

Next, we investigated the relationship between propulsion asymmetry magnitude and CGAM at baseline. To do this, we fit a robust linear mixed-effects model with CGAM as the outcome, a fixed effect for propulsion asymmetry magnitude, and a random intercept for each participant. We fit the model to the final 42 strides of the baseline trial for each participant, which matched the minimum number of strides taken during the baseline trial in the cohort.

To test our primary hypothesis, we examined the relationship between Δ|PA| and the corresponding Δ CGAM. To do this, we fit a robust linear mixed-effects model with Δ CGAM as the outcome, a fixed effect for Δ|PA|, and a random intercept for each participant. We also included a random slope because it improved the model fit. We fit the model to the last 113 strides of each biofeedback trial for each participant, which matched the minimum number of strides per biofeedback trial taken in the cohort.

## RESULTS

Twenty-nine individuals with chronic stroke participated in the study. Two participants were excluded due to marker occlusion, four were excluded because they did not have a propulsion asymmetry > 0.11 at baseline, and two were excluded because they could not consistently generate any paretic propulsion while walking (i.e., paretic propulsion = 0; see Supplemental Results). Therefore, we included 21 participants in the analyses. Clinical demographics for the included participants are provided in Table 1.

### Participants used biofeedback to increase paretic propulsion and reduce propulsion asymmetry

**Figure 1:**
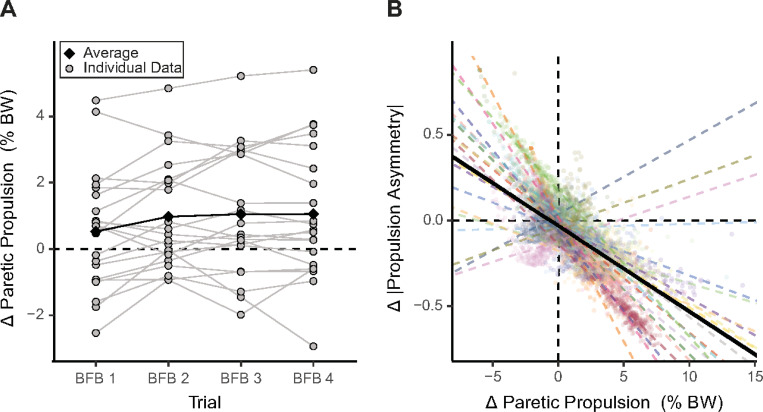
Change in paretic propulsion across trials and the relationship between change in paretic propulsion and change in propulsion asymmetry magnitude. **A)** Average change in paretic propulsion from baseline for biofeedback trials. **B)** Change in paretic propulsion vs. change in propulsion asymmetry. The solid black line is the group-level model fit (fixed effect).

During the biofeedback trials, peak paretic propulsion was within the provided goal zone for 36% of strides, below the goal zone for 51% of strides, and above the goal zone for 13% of strides across all participants. Despite over half of the strides being below the paretic propulsion goal, participants still increased paretic propulsion compared to baseline during biofeedback trials 2–4 (Figure 1A, Table 2). This confirms that participants were able to use the biofeedback to increase paretic propulsion as intended.

We found that increased paretic propulsion was related to reduced propulsion asymmetry magnitude (β = −0.05, p = 0.0001; Figure 1B). On average, an increase in propulsion of 1% BW corresponded to a 5% reduction in propulsion asymmetry. Of note, for this analysis, we only included data from trials 2–4, where participants used biofeedback to successfully increase paretic propulsion.

### Propulsion asymmetry magnitude and CGAM were not related at baseline

**Figure 2:**
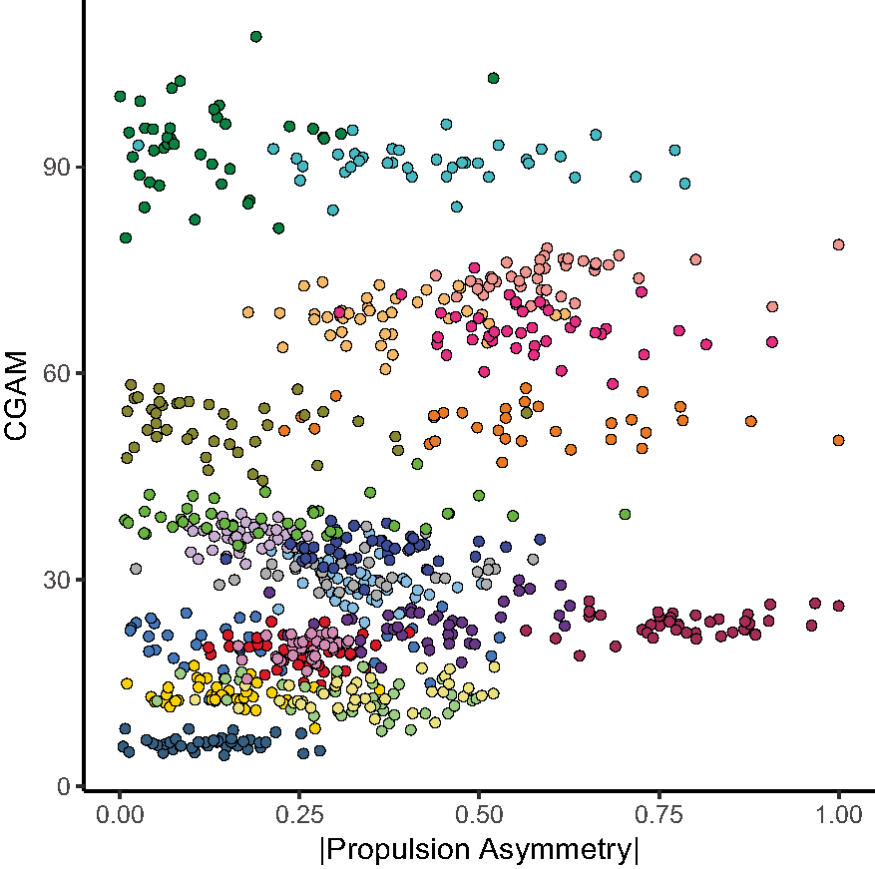
Relationships between propulsion asymmetry magnitude and CGAM at baseline. Each point represents data from an individual stride, colored by participant.

Baseline propulsion asymmetry magnitude was not associated with baseline CGAM (p = 0.7; Figure 2), suggesting that participants with similar levels of baseline propulsion asymmetry had different baseline CGAM. On an individual level, participants tended to have a relatively consistent CGAM across variable propulsion asymmetry magnitudes.

### Changes in propulsion asymmetry magnitude were associated with limited changes in CGAM

**Figure 3:**
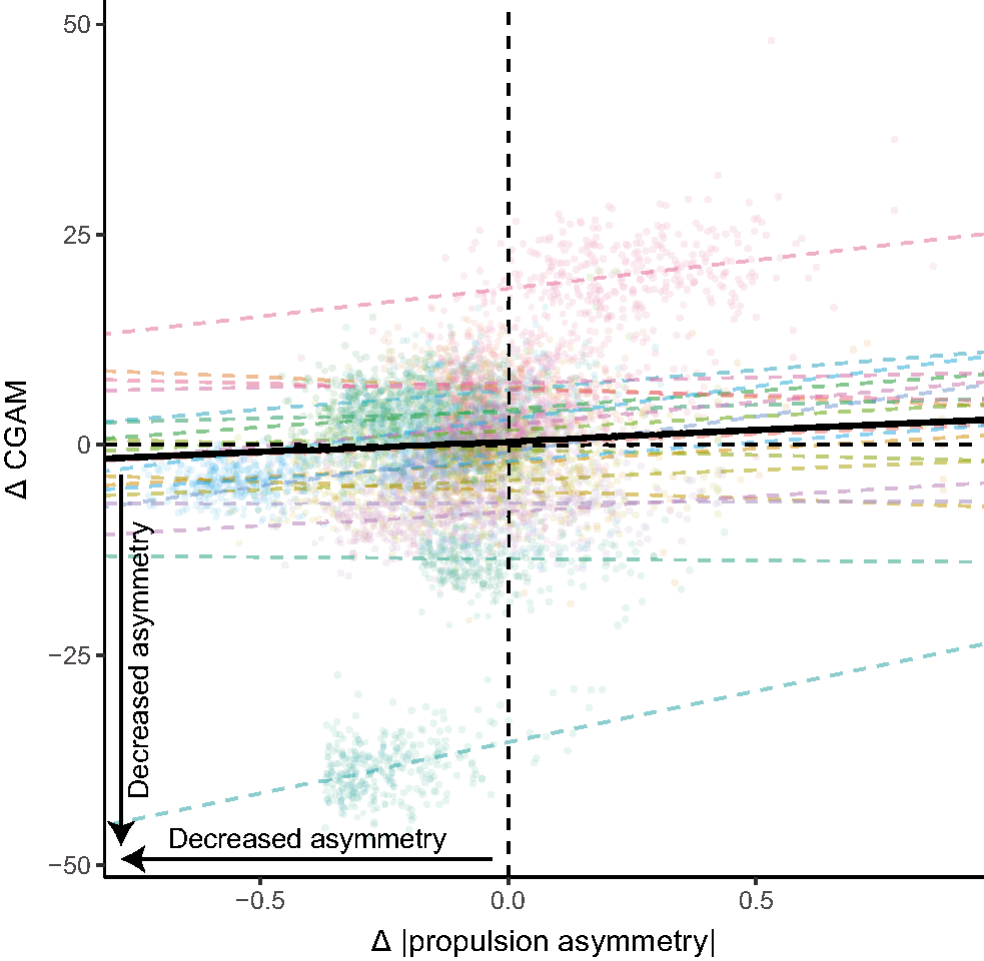
Relationship between change in propulsion asymmetry magnitude and change in the combined gait asymmetry metric (CGAM). The solid black line is the group-level model fit (fixed effect). The dashed lines represent individual model fits from the robust mixed-effects model. Data points are data for an individual stride, colored by participant. Therefore, when baseline propulsion asymmetry is subtracted, the resulting change in propulsion asymmetry was the same for these strides.

We found a significant association between Δ|PA| and Δ CGAM (intercept β = 0.47, p = 0.78; Δ|PA|β=2.6, p = 0.009; Figure 3). However, there was large individual variability across our participants in the relationship between Δ|PA| and Δ CGAM, with some participants demonstrating a stronger association and some showing a weaker association (Figure 3). Additionally, the average change in propulsion asymmetry magnitude in our sample was −0.09 (23% reduction from baseline), suggesting that, on average, we would expect to see a CGAM change of 0.2 (0.5% increase from baseline). Since the average baseline CGAM was 39, this indicates that reducing propulsion asymmetry with biofeedback is unlikely to generate substantial changes in overall gait asymmetry. The relationship between CGAM and Δ|PA| was not impacted by AFO use (p = 0.73), treadmill speed (p = 0.43), or handrail use (p = 0.85). Of note, for this analysis, we only included data from trials 2–4, where participants successfully used biofeedback to increase paretic propulsion.

## Discussion

We aimed to understand how changes in propulsion asymmetry related to changes in overall gait asymmetry, measured by CGAM. We found a significant association between Δ|PA| and Δ CGAM; however, the average expected CGAM change (0.2) suggests that reducing propulsion asymmetry likely will not result in substantial changes to overall gait asymmetry. This suggests that biofeedback-based approaches to reduce propulsion asymmetry may need to be combined with other interventions to improve overall gait asymmetry.

### Participants used biofeedback to increase paretic propulsion and reduce propulsion asymmetry

Consistent with previous work,^7^ we found that participants were able to use paretic propulsion biofeedback to increase paretic propulsion and reduce propulsion asymmetry. Our results indicate the biofeedback paradigm was challenging. Specifically, in only 36% of strides taken across all participants was the peak paretic propulsion produced was sufficient to reach the propulsion goal, whereas, for 51% of strides, the participants’ peak paretic propulsion was below the propulsion goal zone. However, there was still a group-level increase in paretic propulsion with practice, which led to a group-level decrease in propulsion asymmetry. Notably, the strength and direction of the relationship between paretic propulsion and propulsion asymmetry varied across participants. Three participants increased propulsion asymmetry when they increased paretic propulsion (Figure 1B). This may be, in part, because the biofeedback only provided information about paretic limb propulsion. Therefore, participants’ success using the biofeedback did not depend on change in their non-paretic propulsion. Participants could have increased paretic propulsion while decreasing non-paretic propulsion, resulting in increased propulsion asymmetry. Future studies of propulsion biofeedback should consider providing bilateral propulsion biofeedback to ensure increases in paretic propulsion lead to a reduction in propulsion asymmetry across participants.

### Changing propulsion asymmetry within a single session had a limited effect on overall gait asymmetry

Within-session changes in propulsion asymmetry had a limited effect on overall gait asymmetry. This was somewhat surprising due to associations between paretic propulsion and many variables included in our CGAM calculation, such as trailing limb angle,^14^ swing knee flexion,^13^ and step length asymmetry.^3^ A few factors may explain the small relationship between Δ|PA| and Δ CGAM. First, there are multiple degrees of freedom in the lower limb, resulting in various limb configurations that can reduce propulsion asymmetry without reducing overall gait asymmetry. This idea is supported by our baseline data that showed within an individual propulsion asymmetry could vary between strides while CGAM remained relatively stable, and between individuals who took strides with similar propulsion asymmetry values, the associated CGAM varied widely (Figure 2). The number of possible limb configurations used to generate paretic propulsion may have been larger in our study due to providing only paretic propulsion biofeedback, leaving the nonparetic limb unconstrained.

Secondly, the muscles recruited to increase paretic propulsion (and reduce propulsion asymmetry) may have an undesirable effect on overall gait asymmetry. Propulsion asymmetry is inversely associated with paretic plantar flexor activity.^28^ However, individuals post-stroke can still generate relatively high levels of paretic propulsion without plantar flexor activity by recruiting the hamstrings to compensate.^28^ Recruiting the hamstrings instead of the plantar flexors to reduce propulsion asymmetry may lead to undesirable changes in other biomechanical variables. Musculoskeletal simulation work by Sauder et al.^29^ found that optimizing functional electrical stimulation to the calf while walking reduced propulsive asymmetry. Yet, the resulting reduction in propulsion asymmetry did not restore symmetry in kinematic measures (e.g., knee flexion, hip flexion).^29^ Additionally, inappropriate recruitment of musculature while walking can also increase braking forces,^30^ which are associated with reduced swing knee flexion,^28,31,32^ reduced trailing limb angle,^32^ and increased step length asymmetry,^33^ and may impact overall gait asymmetry. Future work should incorporate electromyography measures to help understand muscular contributions to changes in overall gait asymmetry.

The timing of peak paretic propulsion may also be an important factor contributing to the relationship between propulsion asymmetry and overall gait asymmetry. Peak paretic propulsion occurs earlier in stance phase in individuals post-stroke compared to neurotypical adults, and earlier in the paretic limb than the non-paretic limb.^34^ The timing of paretic propulsion assistance has been related to metabolic cost in individuals post-stroke,^35^ suggesting that propulsion timing plays an important role in post-stroke gait. It is currently unclear how propulsion timing relates to other biomechanical impairments in individuals post-stroke. One limitation to our study design is that we did not control propulsion timing with our biofeedback scheme. Additional work is necessary to understand the effect of both propulsion magnitude and timing on overall gait asymmetry.

Finally, participants only completed a single session of paretic propulsion biofeedback training. It is possible that with practice, individuals may learn a different gait strategy to maximize propulsion that may lead to a more symmetric gait or may increase their capacity to increase propulsion with longer-term training. Evidence shows that five weeks of robotic gait training targeting paretic propulsion increased paretic propulsion and reduced propulsion asymmetry.^36^ This suggests that individuals post-stroke can increase paretic propulsion capacity with repeated training. However, it is currently unclear how repeated paretic propulsion biofeedback training sessions would impact overall gait asymmetry.

### Benefits and limitations of CGAM

We chose to use CGAM as our measure of overall gait asymmetry for multiple reasons. First, CGAM does not require large amounts of normative data (as the Gait Deviation Index and Gait Profile Score do).^37,38^ Second, CGAM can capture kinematic and spatiotemporal impairments simultaneously in a single measure (interlimb asymmetry only captures sagittal plane kinematic impairment)^22^ and allows inclusion of circumduction and timing metrics that are not captured by other asymmetry measures. Finally, CGAM allows for flexible inputs, allowing you to tailor it within the confines of your data set. However, this flexibility limits comparison between CGAM values calculated with different variables.

With the CGAM, you must select which biomechanical impairments to include, which is sensitive to the biomechanical impairments included. We chose to include as many commonly studied biomechanical impairments in the CGAM calculation as possible within the confines of our marker set. However, we were unable to include hip hiking because some participants had negative values (i.e., hip drop), which cannot be used in the CGAM calculation.

### Relationship between overall gait asymmetry clinically relevant measures after stroke

To date, very few studies in adults with stroke have included overall gait asymmetry measures, even though individuals post-stroke often cite improving gait appearance as an important rehabilitation goal.^12^ The importance of assessing overall gait asymmetry is reflected in the most recent recommendations from the third Stroke Recovery and Rehabilitation Roundtable, which recommend including multivariate kinematic measures in research studies.^42^ The intention is to provide a comprehensive and clinically meaningful measure of walking impairment.^42^ However, no work has directly investigated the effect of manipulating overall gait asymmetry on clinically meaningful measures of walking. There is evidence that the Gait Deviation Index is moderately correlated with gait speed and step count^43^ and that CGAM (calculated using step length, step time, swing time, double limb support, and vertical GRF) is moderately correlated with gait distance and speed.^16^ This suggests a potential relationship between multivariate kinematic metrics and activity-level measures, but more work is necessary to delineate the strength of the relationship.

Perhaps because this is not common practice, it remains unclear how overall gait asymmetry relates to other clinically important measures of walking function and impairment and whether reducing overall gait asymmetry is necessary to achieve or maximize progress toward different rehabilitation goals (e.g., faster walking speeds). Asymmetric gait may be necessary to optimize some other aspect of gait. For example, reducing step length asymmetry impairs dynamic balance in individuals post-stroke,^44^ and predictive simulation work suggests that spatiotemporal asymmetry may be metabolically optimal for individuals post-stroke.^45^ Additionally, individuals post-stroke can increase gait speed without improving kinematic symmetry,^46^ suggesting that reducing overall gait asymmetry may not be necessary to improve gait speed. However, no studies have directly investigated the impact of reducing overall gait asymmetry on metabolic cost, stability, or gait speed; therefore, more work is necessary to define the relationship between these domains of walking impairment and overall gait asymmetry.

## CONCLUSIONS

We found that individuals post-stroke were able to use visual biofeedback to increase paretic propulsion and reduce propulsion asymmetry. These reductions in propulsion asymmetry were associated with small changes in overall gait asymmetry. This work provides a more comprehensive understanding of the short-term advantages and limitations of manipulating propulsion using biofeedback after stroke. Biofeedback-based approaches to reduce propulsion asymmetry may need to be combined with other interventions to improve overall gait asymmetry. More work is needed to understand the relationship of CGAM with measures of walking activity.

## Supplementary Material

Supplement 1

## Figures and Tables

**Table 1:** Participant demographics.

Race/Ethnicity	Sex	Age	Affected side	AFO	LEFM	Berg	6-minute walk test (m)	10-meter walk speed (m/s)	Treadmill speed (m/s)
Asian	M	63	R	No	28	51	378	1.19	0.61
Asian	F	53	R	No	29	49	322	1.07	0.55
White	M	59	L	No	29	45	221	0.70	0.37
White/Hispanic	F	35	R	No	24	45	424	1.39	0.78
White/Hispanic	M	61	L	Yes	22	42	223	0.73	0.39
Asian	M	59	L	No	24	50	370	1.19	0.74
White	M	74	L	No	26	44	104	0.37	0.25
White	F	61	R	No	24	52	162	0.51	0.50
Asian	M	53	R	No	25	52	431	1.17	0.60
Black	M	66	R	No	27	49	266	1.54	0.84
Asian	M	78	L	No	24	48	236	0.86	0.49
White/Hispanic	F	46	L	Yes	24	52	249	0.95	0.54
White/Hispanic	F	55	L	No	28	55	311	1.04	0.54
White/Hispanic	M	63	L	Yes	18	53	247	0.92	0.56
Black	M	56	L	Yes	11	32	243	0.81	0.52
White	F	64	R	Yes	23	46	315	0.92	0.48
White/Hispanic	M	69	L	Yes	22	39	98	0.23	0.30
Black	M	79	L	No	28	50	431	1.38	0.72
White/Hispanic	M	44	R	Yes	16	54	428	1.07	0.58
White/Hispanic	F	31	L	No	25	55	280	0.86	0.46
Asian	M	37	R	Yes	22	49	109	0.26	0.20

Abbreviations: LEFM, Lower Extremity Fugl-Meyer; M, male; F, female; R, right; L, left.

**Table 2.** Average paretic propulsion and change in paretic propulsion across trials.

	Paretic Propulsion (% BW)	Δ Paretic Propulsion (% BW)
Baseline	5.6 (2.4)	
Trial 1	6.2 (2.4)	0.5 (1.8)
Trial 2	6.6 (2.5)*	1.0 (1.6)
Trial 3	6.7 (2.4)*	1.1 (1.9)
Trial 4	6.7 (2.4)*	1.1 (2.0)

Abbreviations: % BW, percent body weight

## Data Availability

The datasets used and/or analyzed during the current study are available from the corresponding author upon reasonable request.
