# Supplementary material for: Within-session propulsion asymmetry changes have a limited effect on gait asymmetry post-stroke
Source: Res Sq. 2024 Dec 23:rs.3.rs-5053605. Preprint. [Version 1] doi: 10.21203/rs.3.rs-5053605/v1 (PMC11703335; doi:10.21203/rs.3.rs-5053605/v1)
Supplement: Supplement 1 [file NIHPPRS5053605v1-supplement-1.pdf]

Supplementary Files

This is a list of supplementary files associated with this preprint. Click to download.

- SupplementalMaterials.pdf
